# Knowledge gaps hamper understanding the relationship between fragmentation and biodiversity loss: the case of Atlantic Forest fruit-feeding butterflies

**DOI:** 10.7717/peerj.11673

**Published:** 2021-06-25

**Authors:** Thadeu Sobral-Souza, Juliana Stropp, Jessie Pereira Santos, Victor Mateus Prasniewski, Neucir Szinwelski, Bruno Vilela, André Victor Lucci Freitas, Milton Cezar Ribeiro, Joaquín Hortal

**Affiliations:** 1 Departamento de Botânica e Ecologia, Universidade Federal de Mato Grosso, Cuiaba, Mato Grosso, Brazil; 2 Department of Biogeography and Global Change, Museo Nacional de Ciencias Naturales (MNCN-CSIC), Madrid, Spain; 3 Instituto de Ciências Biológicas, Universidade Federal de Alagoas, Maceio, Brazil; 4 Departamento de Biologia Animal, Universidade Estadual de Campinas, Campinas, Brazil; 5 Programa de Pós-Graduação em Ecologia e Conservação da Biodiversidade, Universidade Federal de Mato Grosso, Cuiabá, Brazil; 6 Laboratório de Orthropterologia, Universidade Estadual do Oeste do Paraná, Cascavel, Brazil; 7 Universidade Federal da Integração Latino Americana, Foz de Iguaçu, Paraná, Brazil; 8 Instituto de Biologia, Universidade Federal da Bahia, Salvador, Brazil; 9 Instituto de Biociências, Universidade Estadual de São Paulo, Rio Claro, Brazil; 10 Departamento de Ecologia, Universidade Federal de Goiás, Goiânia, Goiás, Brazil

**Keywords:** Biodiversity data, Deforestation, Butterflies, Habitat fragmentation, Atlantic Forest, Landscape, Macroecology, Sampling bias

## Abstract

**Background:**

A key challenge for conservation biology in the Neotropics is to understand how deforestation affects biodiversity at various levels of landscape fragmentation. Addressing this challenge requires expanding the coverage of known biodiversity data, which remain to date restricted to a few well-surveyed regions. Here, we assess the sampling coverage and biases in biodiversity data on fruit-feeding butterflies at the Brazilian Atlantic Forest, discussing their effect on our understanding of the relationship between forest fragmentation and biodiversity at a large-scale. We hypothesize that sampling effort is biased towards large and connected fragments, which occur jointly in space at the Atlantic forest.

**Methods:**

We used a comprehensive dataset of Atlantic Forest fruit-feeding butterfly communities to test for sampling biases towards specific geographical areas, climate conditions and landscape configurations.

**Results:**

We found a pattern of geographical aggregation of sampling sites, independently of scale, and a strong sampling bias towards large and connected forest fragments, located near cities and roads. Sampling gaps are particularly acute in small and disconnected forest fragments and rare climate conditions. In contrast, currently available data can provide a fair picture of fruit-feeding butterfly communities in large and connected Atlantic Forest remnants.

**Discussion:**

Biased data hamper the inference of the functional relationship between deforestation and biodiversity at a large-scale, since they are geographically clustered and have sampling gaps in small and disconnected fragments. These data are useful to inform decision-makers regarding conservation efforts to curb biodiversity loss in the Atlantic Forest. Thus, we suggest to expand sampling effort to small and disconnected forest fragments, which would allow more accurate evaluations of the effects of landscape modification.

## Introduction

Species are disappearing worldwide at an alarming rate, particularly in tropical regions (Dirzo & Raven, 2003) where extinction and defaunation processes result from anthropogenic environmental impacts (Crutzen, 2006; Ceballos et al., 2015; Dirzo et al., 2014). In the Neotropics, species extinction is directly related to deforestation (Estrada et al., 2016). However, an in-depth understanding of how deforestation causes biodiversity loss for specific taxa in relation to different levels of landscape fragmentation is still missing (Chase et al., 2020).

Assessing the vulnerability of species to extinction caused by deforestation needs long-term data on population dynamics at different spatial scales and levels of landscape fragmentation (Fahrig, 2017). However, community data documenting the species abundance and richness for different localities, specifically in fragmented landscapes, are scarce (Martin, Blossey & Ellis, 2012). The lack of such data curbs making reliable assessments of population trends through time and along different gradients of landscape fragmentation. In this sense, extensive on-line biodiversity information could help us to understand species extinctions, correlating changes in species occurrence with deforestation and habitat fragmentation. However, information about species occurrence and distributions available in on-line databases is potentially biased (Hortal, Lobo & Jiménez-Valverde, 2007; Hortal et al., 2008; Lobo et al., 2007; Boakes et al., 2010; Stropp et al., 2016; Stropp et al., 2020). Such biases in species occurrence data could be partly addressed by adding the records of species occurrence from small natural history collections and scientific literature that are typically not present in on-line databases (Robertson et al., 2014; Gries, Gilbert & Franz, 2014; Geijzendorffer et al., 2015; Soltis & Soltis, 2016). Data mobilization (i.e., making data available for use by anyone and anywhere) can partially reduce the spatial and temporal bias, and increase the predictive power of models, allowing assessing ecological patterns accurately (see Whittaker et al. (2005)). Nevertheless, the use of the assembled big data can potentially add noise and/or bias into conservation studies. This issue naturally stems from pulling together data gathered using different survey methods and collected for very different specific questions, which may or may not be related to the aims of the new big data studies. As far as we know, the extent to which mobilizing biodiversity big data allows studying the functional relationship between forest fragmentation and large-scale biodiversity loss has not been subject to systematic evaluation yet.

The Brazilian Atlantic Forest is a highly human-impacted biodiversity hotspot, which is threatened by deforestation, climate change, and invasive species (Mittermeier et al., 2005; Bellard et al., 2014; Joly, Metzger & Tabarelli, 2014; Bello et al., 2020). The increase in forest fragmentation, with the consequent increment in the number of small forest patches (Taubert et al., 2018), translates into an overall loss of biodiversity and changes in ecosystem functioning (Sala et al., 2000; Dirzo & Raven, 2003; Butchart et al., 2010; Haddad et al., 2015; Chase et al., 2020; Hortal & Santos, 2020). The Atlantic Forest fragmentation is the main driver of the loss of mammals (Pardini, 2004; Galetti et al., 2009; Jorge et al., 2013), birds (Metzger et al., 2009; Boscolo & Metzger, 2009), reptiles (Gibbons et al., 2000; Almeida-Gomes & Rocha, 2014a), amphibians (Almeida-Gomes & Rocha, 2014b; Almeida-Gomes et al., 2016), dung beetles (Alves & Hernández, 2019), other invertebrates (Didham et al., 1996), and plants (Hobbs & Yates, 2003). In the same way, Atlantic Forest fragmentation caused the depletion of ecosystem functions such as pollination (Hadley & Betts, 2011; Jaffé et al., 2016) and seed dispersal (Galetti, Alves-Costa & Cazetta, 2003; Galetti et al., 2006). Most of these studies are based on small-scale data from a few forest fragments, but their outcomes are commonly used to infer the overall impact of forest fragmentation on biodiversity and ecosystem function at large scale. The scarcity of standardized biodiversity inventories across large geographical areas mostly explains the lower proportion of large-scale studies in comparison to local studies. Yet, the publication of data papers has been contributing to fill this knowledge gap (Galetti & Ribeiro, 2018). Besides these compilation efforts, the study of the spatial and environmental sampling bias in the knowledge of a taxon is also a crucial challenge to improve the understanding of the fragmentation effects on biodiversity across different spatial scales.

Here we assess the sampling coverage biases and gaps in fruit-feeding butterfly biodiversity data, discussing the possibility to infer or not the relationship between deforestation and the loss of diversity from this group at large scale in the Brazilian Atlantic Forest. We focus our study on fruit-feeding butterflies (Lepidoptera) whose occurrence is sensitive to environmental conditions (Brown & Freitas, 2000; Bonebrake et al., 2010). The fruit-feeding butterflies are mostly used to assess the effects of forest fragmentation as they respond quickly to changes in environmental conditions, are easy to capture using standardized sampling traps, and their taxonomic identification is straightforward compared to other insect groups (Freitas et al., 2014). The fruit-feeding butterfly communities of the Atlantic Forest have been surveyed across several localities for around fifty years. Most of this information was until recently restricted to field guides or to the sampling made by naturalists, who did field expeditions to collect as many species as possible mainly in forest sites and protected areas, typically located at large forest remnants. The recent digitalization of such information allowed the construction of several datasets, whose quality relies on an extensive compilation of standardized samplings and field observations conducted over 54 years across 122 localities (see Santos et al., 2018).

We have used this new dataset to investigate and quantify spatial biases and gaps in the occurrence of fruit-feeding butterflies from the Brazilian Atlantic Forest. We seek to assess how previous sampling effort affects our capacity to assess the functional relationship between deforestation and trends in fruit-feeding butterflies at large scale in this biome. We hypothesize that sampling efforts are spatially biased towards large and connected fragments, which occur geographically clustered in the Atlantic forest nowadays. We predict that these biases prevent inferences of relation between fragmentation and biodiversity loss at large scale.

## Materials & Methods

### Study area

**Figure 1:**
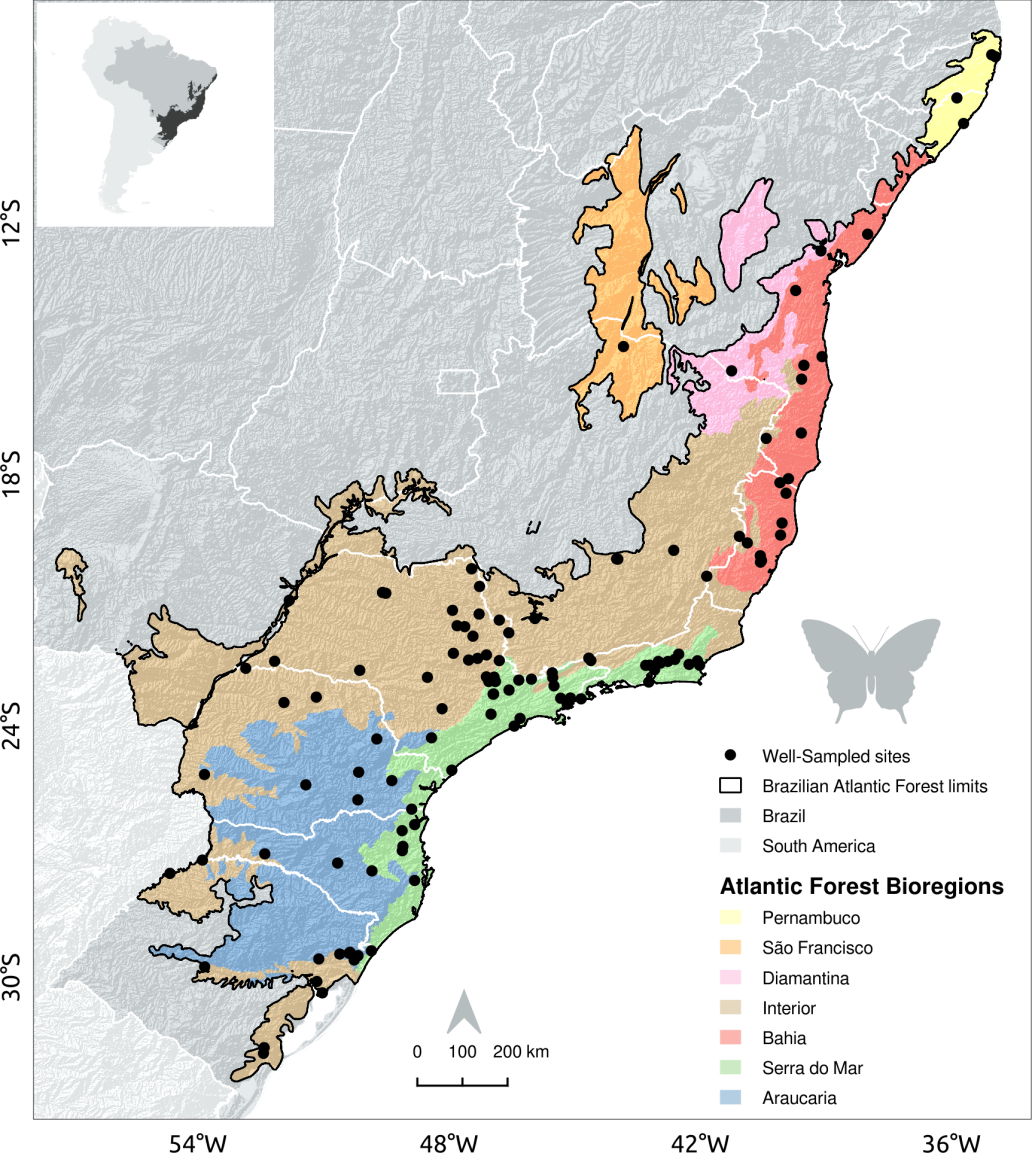
Location of sites with well-sampled fruit-feeding butterfly inventories in the Brazilian Atlantic Forest. Black dots indicate well-sampled sites of fruit-feeding butterflies; White lines mark the limits of Brazilian states; dark gray areas highlight forest remnants and background colour shades show the Brazilian Atlantic Forest bioregions proposed by Silva & Casteleti (2003).

The Brazilian Atlantic Forest stretches from the southern to the northeastern Atlantic coast of Brazil (see Ribeiro et al. (2009), Fig. 1). Two main forest types prevail: a dense tropical rainforest occurs close to the Atlantic coast; a seasonal forest occurs inland at altitudes higher than 600 m above sea level. The Brazilian Atlantic forest biome comprises five bioregions (Bahia, Brejos Nordestinos, Pernambuco, Diamantina, and Serra do Mar), and three regions that are considered transition zones (São Francisco Forest, Araucaria Forest, and Interior Forests) (Silva & Casteleti, 2003; Fig. 1). Despite its high species richness and endemism, the original Atlantic Forest landscape has been severely modified, with the remaining mosaic of small and disconnected forest fragments accounting for less than 12% (150 million hectares) of the original forest cover (Ribeiro et al., 2009). About 80% of these fragments are smaller than 50 hectares (Joly, Metzger & Tabarelli, 2014) and poorly connected to larger forest remnants (Ribeiro et al., 2009).

### Species data

All fruit-feeding butterflies occurring in the Atlantic forest belong to the family Nymphalidae forming a large polyphyletic group within this family. The larvae are herbivorous and adults feed on decomposing fruits and other fluids like plant exudates or mammal excrements (Devries, 1987; Freitas et al., 2014). We obtained records of species occurrence from two on-line biodiversity databases: Global Biodiversity Information Facility (GBIF, 2020 DOI https://doi.org/10.15468/dl.bnbeqo) and SpeciesLink (2020).

Besides these records, we added all records compiled by Santos et al. (2018), who made an exhaustive compilation of data on local checklists covering 54 years of continuous sampling and observations. This dataset includes: (1) all local species checklists recorded by Prof. K. Brown Jr. from 1964 to 2006 and by Prof. A. V. L. Freitas from 1988 to 2018, and (2) local checklists recorded in studies published in peer-reviewed journals, PhD, Masters and BSc theses, and technical reports). These checklists contain records compiled in exhaustive local inventories of areas typically smaller than 1 km^2^. In total, Santos et al. (2018) dataset contains records on 279 fruit-feeding butterfly species.

We then combined all records gathered from these three sources (i.e., GBIF, SpeciesLink and Santos et al., 2018) into a single dataset. We excluded duplicate records, i.e., records of the same species that were collected at identical latitude, longitude and date. We also excluded records which: (1) geographic coordinates were given with a precision of less than three decimal places (i.e., approximately 100 m of spatial precision) to ensure observations can be attributed also to small forest fragments; (2) coordinates placed outside Brazil; and (3) coordinates coinciding with the center of a city/village and/or museum collections (i.e., populated places and places classified as administrative level 2 according to GEONAMES, 2020) (https://www.geonames.org/). Additionally, all records holding erroneous scientific names, except accepted synonyms, were excluded from our analyses following Lamas (2004) as correct taxonomy data. Finally, we selected from this dataset only records belonging to the 279 species listed by Santos et al. (2018) because this dataset contains taxonomically revised names with updated taxonomy.

### Climate variables and landscape metrics

We used the 19 bioclimatic variables available in WorldClim 2.0 database (Fick & Hijmans, 2017) at 1 km^2^ resolution to characterize the climatic conditions of Atlantic Forest. These variables were downloaded in worldwide extension and then clipped to the limit of the Brazilian Atlantic forest (Ribeiro et al., 2009). Then, we applied a Principal Component Analysis (PCA), with varimax rotation, to obtain the main climatic gradient across the Atlantic forest. The first two axes of the PCA summarize the variation of climate in the Atlantic forest and constitute our definition of climate environmental space, in 0.25 × 0.25 km resolution, for all subsequent analyses.

We used five landscape metrics to characterize the spatial features of Atlantic Forest patches: (1) proportion of forest cover; (2) patch size; (3) homogeneity of forest cover; (4) 200 m functional connectivity—as a proxy for butterfly dispersal ability; and (5) Euclidean distance from roads and urban areas. We used these metrics because they capture the structure and configuration of forest patches that affect the composition of butterfly communities (Santos et al., 2020). All landscape metrics were calculated for geospatial grid cells of 1 km^2^ cell-size resolution (Table 1) and are based on maps of natural forest cover produced by the Brazilian Foundation for Sustainable Development (FBDS, 2020), Fundação SOS-Mata Atlântica and the National Institute for Spatial Research (SOS Mata Atlntica Foundation, 2020), and the Global Forest Change project (Hansen et al., 2013). To characterize the spatial features of forest patches, we used maps of non-forest natural vegetation (e.g., Savanna), water bodies, planted forests (silviculture), urban areas, pasture, sugarcane plantations, roads, and other anthropic uses (e.g., other crops, degraded soil, burned area). Moreover, we estimate the anthropogenic influence in the forest patches by calculating the Euclidean distance between forest patches to the nearest road and city. Finally, we performed a Principal Component Analysis (PCA) and used the first two axes of the PCA to summarize the spatial features of forest patches of the Atlantic forest in grid cells of 0.25 × 0.25 km in environmental space.

**Table 1:** Landscape metrics used to describe variations in landscape features, for the assessment of environmental coverage and bias in the information on fruit-feeding butterflies in the Atlantic Forest.

**Metric**	**Description**	**Category**	**Reference**
Proportion of habitat	Proportion of forest habitat within a square window 1 x 1 km, centered in the focal cell (= amount of vegetation cells/total number of cells in the window). It varies between 0 and 100%	Landscape modification	Ribeiro et al. In prep.
Fragment size	Size of the forest fragment where the sampling site was located in (log10 ha)	Landscape modification	Ribeiro et al. In prep.
Functional Connectivity	Area of functionally connected forest (log10 ha * 100) with distance between separate patches ≤ 180 m. Sampling sites located at 180 m far from the forest edge obtain the value as if located inside the fragment	Landscape modification	Ribeiro et al. In prep.
Homogeneity	Similarity of Enhanced Vegetation Index (EVI) between adjacent pixels based on the textural features of EVI. Images were acquired by the Moderate Resolution Imaging Spectroradiometer (MODIS)	Landscape modification	Tuanmu & Jetz (2015)
Distance to urban areas	Euclidean distance to urban area (m)	Anthropogenic	Ribeiro et al. In prep.
Distance to roads	Euclidean distance to nearest road (m)	Anthropogenic	Ribeiro et al. In prep.


### Analysis of inventory completeness

Inventory completeness was calculated for all grid cells of 1 km^2^ resolution of the Brazilian Atlantic Forest, as defined by Ribeiro et al. (2009). For each grid cell, we considered the cumulative number of specimens and species collected only from GBIF and SpeciesLink. Inventory completeness was estimated by the ratio between the number of observed and predicted species, with the latter being obtained from species accumulation curves (Lobo et al., 2018). We obtained the number of predicted species for each cell from the asymptotic value of the sample-based species accumulation curve fitted with the function “Clench” from the R package KnowBR (Lobo et al., 2018), where each record from either GBIF or SpeciesLink was considered as an independent sample (see Hortal & Lobo, 2005). We considered cells as well-sampled when either: (1) they contain more than 50 records and show an inventory completeness higher than 0.7; or (2) they host a local inventory from the list of Santos et al. (2018). Since the dataset compiled by Santos et al. does not contain the information on the number of observations for each individual species, we assumed that each local inventory that passed their scrutiny was complete enough so as to consider it as well-sampled–and by extension the 1 km^2^ grid cell where it is placed. However, this dataset is one of the most comprehensive compilations of sampling and field observations of butterfly communities in the Atlantic Forest (Santos et al., 2020). Thus, the final set of well-sampled sites includes both the results of inventory completeness obtained from on-line biodiversity information databases, and the complete inventories included in Santos et al. (2018) (Table S1).

### Analysis of spatial coverage and bias of well-sampled sites

First we conducted an Average Nearest Neighbor analysis (ANN) with the aim of assessing if the well-sampled sites were spatially overdispersed or aggregated (i.e., clustered) at both narrow and broad geographical scales (i.e., small and large neighbor distances) testing against a null model that include the 117 points randomized 1000 times through the Atlantic Forest delimitation, using 1 km^2^ cell-size resolution. To access the percentage of aggregated sites we calculated the spatial dispersion of sites based on randomly expected mean nearest neighbour distance, thus distances lower than random were considered as aggregated and higher than random as overdispersed.

In addition, we assessed whether well-sampled grid-cells cover the climate and landscape conditions of the Brazilian Atlantic Forest. To this end, we first calculated the frequency of each climate and landscape condition (Table 2) in the environmental space defined by all 0.25 ×  0.25 km grid cells of the biome. Second, we quantified the overlap between the distributions of well-sampled cells and all cells in these two environmental spaces (both climate and landscape) through Schoener’s D index (Schoener, 1970; Warren, Glor & Turelli, 2008). This index characterizes the congruency (i.e., overlap) between two spatial layers varying from zero (total lack of congruency) to one (total congruence) (Broennimann et al., 2012). In our analysis, D values close to one indicate that well-sampled cells are located in most if not all climate and landscape conditions found in the Atlantic forest, while D values close to zero indicate incomplete coverage of climate and landscape conditions, and thus biases in coverage (see Ronquillo et al., 2020 for a similar approach). We tested the significance of D values by comparing the observed D value against a null distribution, generated by drawing 1,000 random samples of 117 cells (i.e., the number of well-sampled cells; see below), and then calculating the Schoener’s D index in each iteration for climate and landscape conditions separately.

**Table 2:** Summary results of the analysis of the coverage, bias and rarity in the description of climate and landscape structure variations across the Atlantic Forest provided by the sites with well-sampled cells fruit-feeding butterfly communities.

Climate		Sampling Coverage		Bias				Rarity	
Bioregions	Number of well-sampled cells	Schoener’s D	Coverage Surface	PC1 *χ*^2^	*p*	PC2 *χ*^2^	*p*	*χ*^2^	*p*
Atlantic Forest	117	0.44	0.24	1.386	0.239	1.377	0.24	37.03	<0.0001^**^
Bahia	16	0.21	0.043	1.089	0.267	2.386	0.122	20.66	<0.0001^**^
Serra do Mar	42	0.22	0.11	1.634	0.201	1.594	0.206	19.29	<0.0001^**^
Pernambuco	4	0.02	0.015	2.554	0.110	1.378	0.240	0.7	0.4
Araucaria	12	0.06	0.034	1.674	0.196	0.250	0.616	3.49	0.06
Interior	41	0.27	0.1	1.824	0.177	0.034	0.855	24.4	<0.0001^*^
Diamantina	2	0.01	0.006	0.004	0.945	0.58	0.446	2.4	0.12

Landscape		Sampling Coverage		Bias				Rarity	
Bioregions	Number of well-sampled cells	Schoener’s D	Coverage Surface	PC1 *χ*^2^	*p*	PC2 *χ*^2^	*p*	*χ*^2^	*p*
Atlantic Forest	117	0.38	0.1	10.39	<0.0001^**^	93.68	<0.0001^**^	113.25	<0.0001^**^
Bahia	16	0.07	0.032	3.301	0.069	0.922	0.337	13.4	<0.0001^**^
Serra do Mar	42	0.19	0.072	16.145	<0.0001^**^	11.941	0.001^*^	28.75	<0.0001^**^
Pernambuco	4	0	0.007	1.224	0.269	2.956	0.086	0.2	0.65
Araucaria	12	0.12	0.035	6.906	0.009^*^	5.629	0.018^*^	19.08	<0.0001^**^
Interior	41	0.36	0.035	11.358	<0.001^**^	51.954	<0.0001^**^	66.2	<0.0001^**^
Diamantina	2	0.01	0.004	4.809	0.028^*^	0.823	0.364	3.75	0.05^*^

**Notes.**

Number of well-sampled cells indicates the number of well-sampled sites. Significant results of the Kruskall-Wallis tests used to assess bias in sampling and rarity sites are marked with ^∗^*p* < 0.05; ^∗∗^*p* < 0.0001.

To assess the coverage of climate and landscape conditions of the Atlantic forest by well-sampled cells, we applied Kruskal-Wallis tests. These tests allowed verifying the extent to which well-sampled cells: (1) provide a representative subset of the overall climate and landscape variation; and (2) cover adequately regions of rare climate and landscape conditions (see similar approaches in Kadmon, Farber & Danin, 2004; Hortal & Lobo, 2011). For this latter purpose, we standardized values of each PCA axis to vary between 0 and 1, where values near one represent rare climate and landscape conditions–that is, environmental conditions that occur in unique grid cells or sites. In contrast, values close to zero represent the most common conditions–climate and landscape conditions that occur in many sites of the Atlantic Forest.

Our analyses considered climate and landscape separately and were performed for the entire Brazilian Atlantic Forest, and separately for each bioregion (see study area section). Only bioregions with more than one well-sampled cell were analyzed. All analyses were performed in R (R Development Core Team, 2018).

## Results

In Santos et al. (2018) we found 6,840 occurrences of fruit-feeding butterflies, recorded in 119 grid-cells, 110 of them located in the Brazilian Atlantic Forest. In GBIF and SpeciesLink we found a total of 411,795 Nymphalidae occurrences, being that 10,672 of them were within the boundaries of the Brazilian Atlantic Forest, and of those 2,201 were from fruit-feeding butterfly species according to Santos et al. (2018). These records were distributed across 75 grid-cells. Only seven grid-cells showed high inventory completeness, i.e., had more than 50 records and an indicator of inventory completeness larger than 0.7. Therefore, we found 117 well-sampled sites: 110 local checklists documented by Santos et al. (2018), and seven additional sites presented high inventory completeness according to GBIF and SpeciesLink records (Fig. 1).

**Figure 2:**
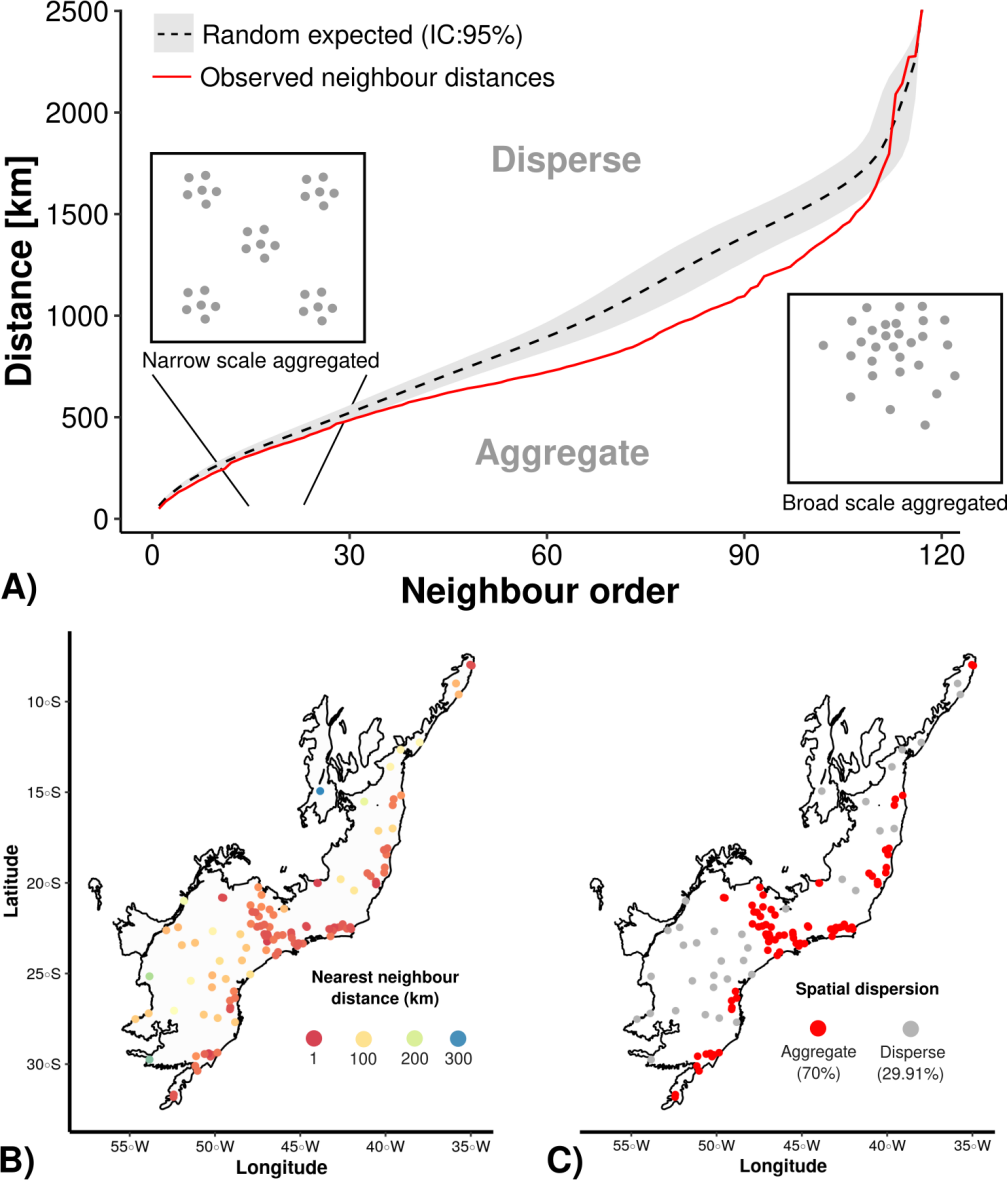
Average Nearest Neighbor analysis showing: (A) the results of randomly expected (in black and grey) and well-sampled sites (red line); (B) well-sampled sites are more aggregated (70%) than (C) overdispersed (30%) independently of scale (nearby or distant neighbour order).

The Average Nearest Neighbor analysis (ANN) indicated that there is an spatial aggregation of well-sampled sampling sites in relation to the null models (Fig. 2). Still, regardless of the scale (nearby or distant neighbors–*X*-axis in Fig. 2A), the sampling data is more aggregated (70% of our sampling sites) than the null model (Fig. 2B, C). Once the results showed a strong geographical sampling bias, further analyses are required to identify the environmental factors (accessibility and/or landscape configuration) that may have caused these spatial biases.

The PCA performed with climate variables did not reveal any strong climate gradient (Fig. S1A). However, in the PCA performed with landscape metrics we identified two main gradients, reflecting mainly variation in the structure of forest cover (Axis 1) and the anthropogenic influence (distances) in the landscape (Axis 2) (Fig. S1B). The first axis, accounting for 45% of the variation, was associated with the proportion of forest cover, patch size, and 200 m functional connectivity (Fig. S1A). The second axis accounted for 34% of the variation and was correlated to distance to cities, roads, and landscape homogeneity (Fig. S1B).

**Figure 3:**
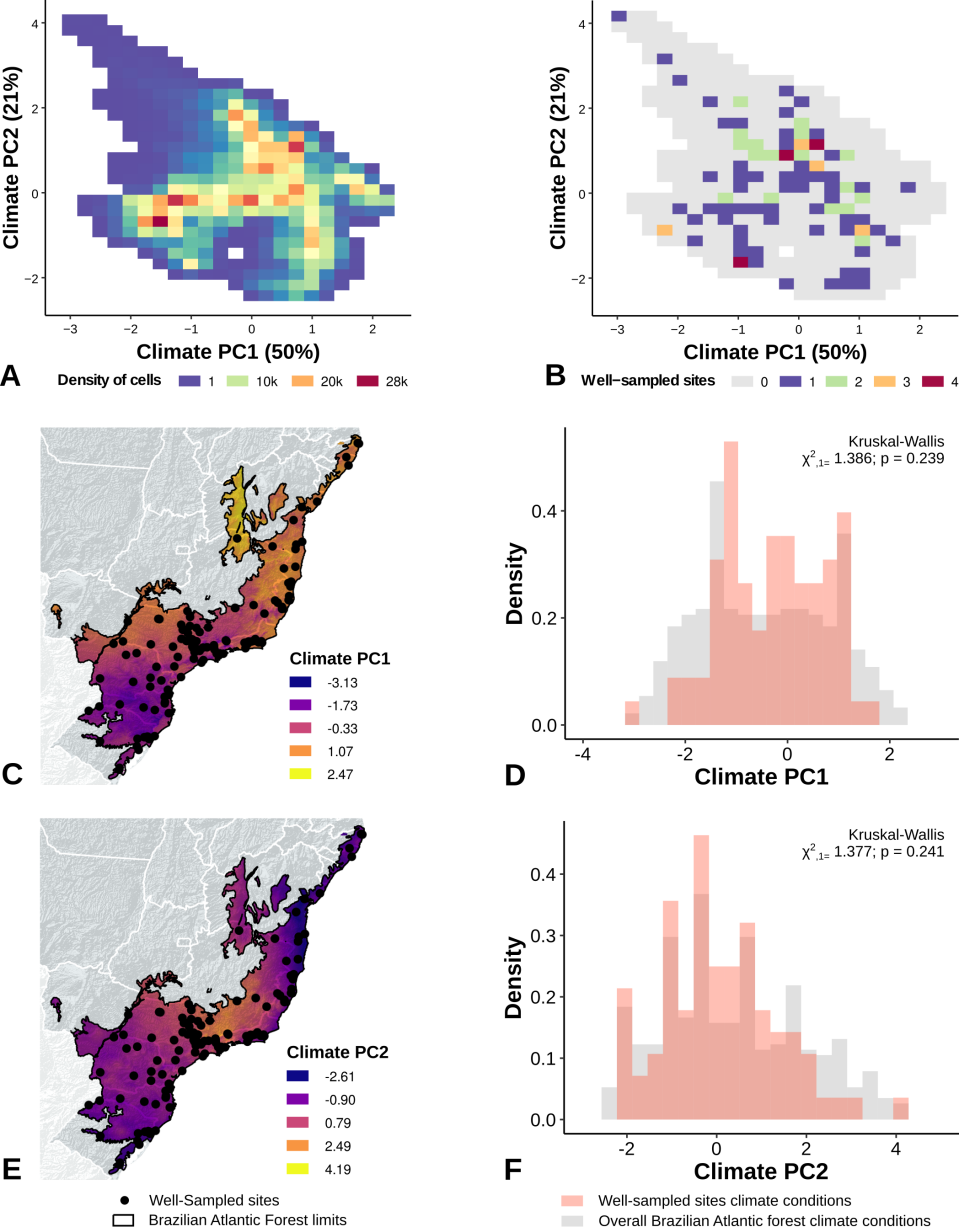
Spatial coverage and bias of sites with well-sampled fruit-feeding butterfly inventories, in relation to the overall climate conditions in the Brazilian Atlantic Forest (A, B). Bias is expressed by density of well-sampled grid in relation to overall Brazilian Atlantic Forest climate condition expressed by PCA axis (PC1, PC2) (C, D, E, F). See ‘Methods’ section and Fig. S1 for more details.

**Figure 4:**
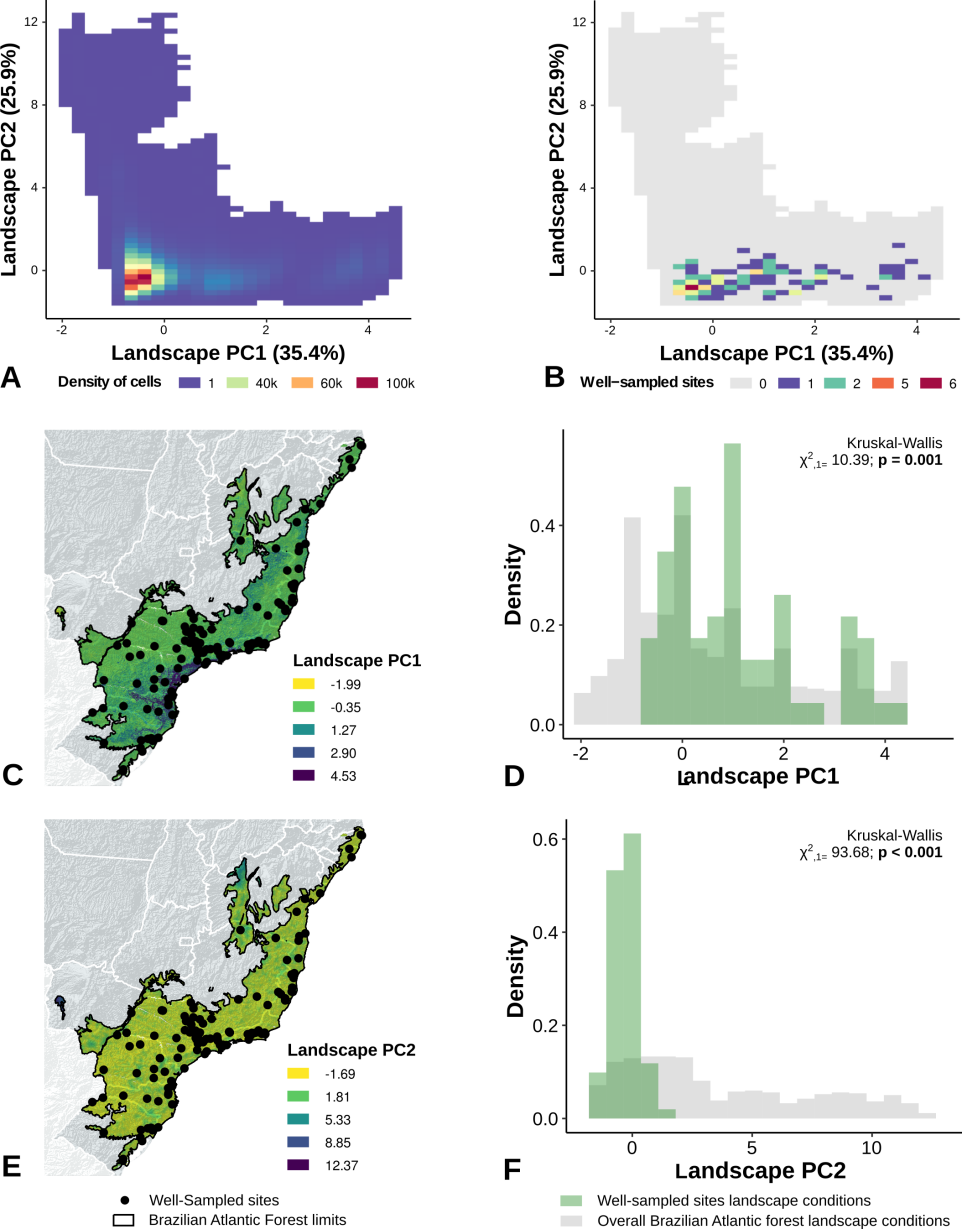
Spatial coverage and bias of sites with well-sampled fruit-feeding butterfly inventories, in relation to the overall landscape conditions in the Brazilian Atlantic Forest (A, B). Bias is expressed by density of well-sampled grid in relation to overall Brazilian Atlantic Forest landscape condition expressed by PCA axis (C,D,E,F) (PC1, PC2). See ‘Methods’ section and Fig. S1 for more details. Note the low coverage of landscape features, highly-biased towards large and connected fragments placed close to cities and roads (see text).

**Figure 5:**
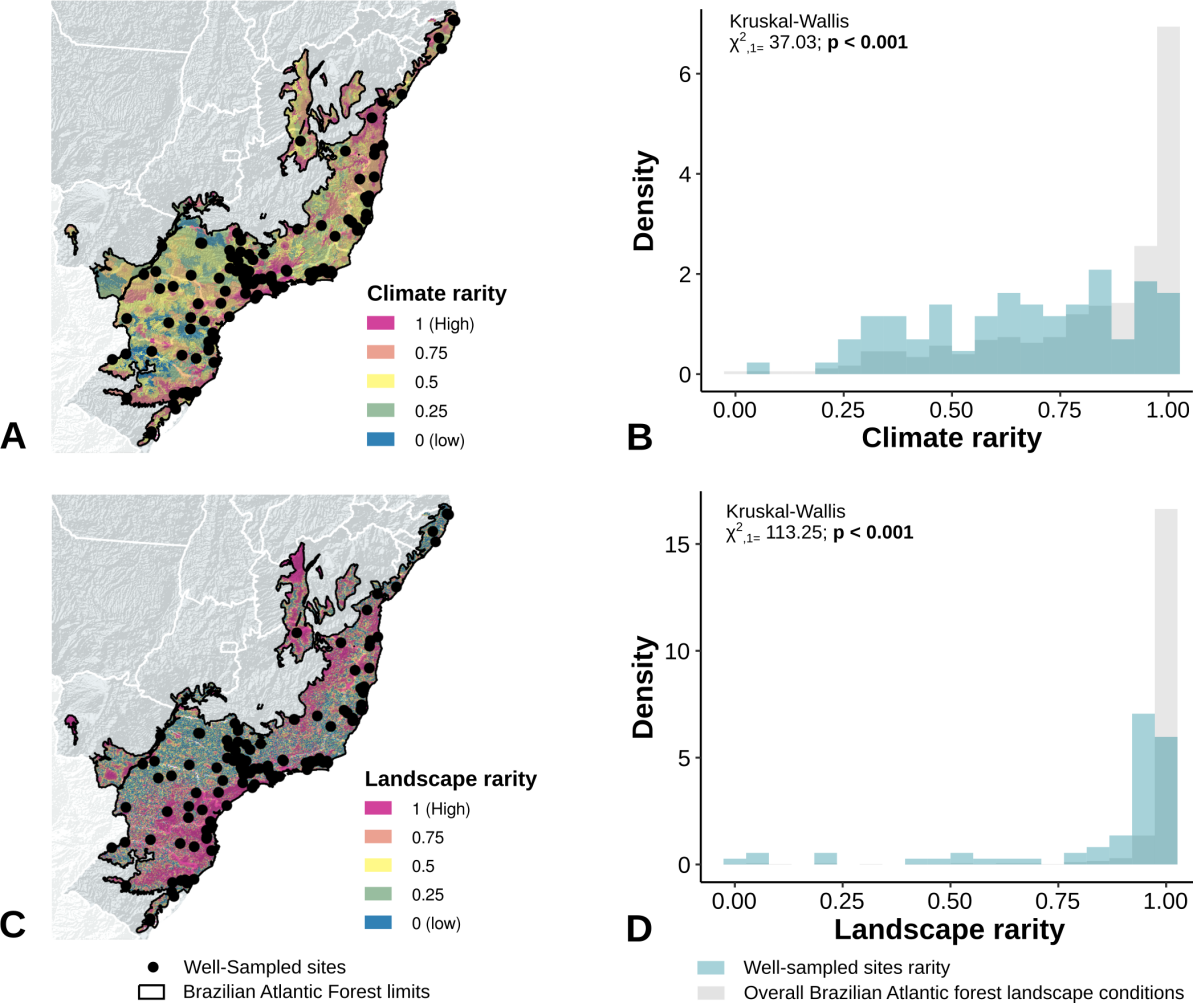
Bias in the location of the sites with well-sampled fruit-feeding butterfly inventories, in relation to rare climate (A and B) and landscape conditions (C and D). Well-sampled sites are highly-biased towards dominant environmental conditions, whereas sampling gaps are acute in rare climate and landscape conditions.

The location of well-sampled cells does not cover all climate and landscape conditions in the Brazilian Atlantic Forest (climate: *D* = 0.44, *p* = 0.001; landscape *D* = 0.38, *p* < 0.001) (Table 3, Figs. 3A and 3B, Figs. 4A and 4B). We found that only 24% and 10% of climate and landscape surfaces, respectively, were well sampled (Table 2). Even covering only 24% of the Atlantic Forest climate surface, the well-sampled cells are not climatically biased (Fig. 3; Table 2; PC1 - *χ*2 = 1.386, *df* = 1, *p* = 0.23; PC2 - *χ*2 = 1.377 *df* = 1, *p* = 0.24). Moreover, we found that the location of well-sampled cells across Atlantic Forest does not capture the whole landscape conditions, but instead is biased towards large and connected forest fragments that are close to cities and roads (Fig. 4, Table 2, PC1: *χ*2 = 10.39, *df* = 1, *p* < 0.0001; PC2: *χ*2 = 93.68, *df* = 1, *p* < 0.0001). Importantly, our results also showed pronounced sampling gaps in rare climate and landscape conditions, (Fig. 5; Table 2, Climate: *χ*2 = 37.03, *df* = 1, *p* < 0.0001; Landscape *χ*2 = 113.25, *df* = 1, *p* < 0.0001) (Fig. 4).

The Interior and Serra do Mar bioregions in the South host a larger number of well-sampled sites than the northern bioregions of the Brazilian Atlantic Forest (Fig. 1, Table 2). Interestingly, the location of well-sampled cells within each bioregion did not present significant climatic biases, but high sampling bias towards large and connected forest fragments, close to cities and roads. Moreover, these regions presented consistent gaps in the coverage of rare climate and landscape conditions, although the interpretation of these results requires some caution due to the low number of sampling sites in each bioregion.

## Discussion

Our findings point that the well-sampled sites of fruit-feeding butterflies are more spatially aggregated than random, stemming from a sampling bias towards large and connected fragments, close to cities and major roads, independently of scale. Besides, we found pronounced survey gaps in rare climate and landscape conditions and oversampling in common conditions—see geographical locations of rare and common climate and landscape conditions (Fig. 5). These results suggest that the current knowledge about the distribution of fruit-feeding butterflies in the Atlantic Forest may be insufficient to infer functional relationships between deforestation and the biodiversity of this group, at least on a broad scale. This highlights the need to both (1) account for these shortcomings (bias) when assessing the responses of fruit-feeding butterflies to forest fragmentation in the Atlantic Forest, and (2) conduct further surveys in a wider range of climate and landscape conditions within this biome.

The spatial biases and gaps in the data on fruit-feeding butterflies reported here are common for insects (Lewinsohn, Freitas & Prado, 2005; Diniz-Filho, De Marco Jr & Hawkins, 2010; Cardoso et al., 2011) and also extend to other taxonomic groups (Lewinsohn & Prado, 2002; Oliveira et al., 2016). A complete assessment of biases and gaps in the spatial and environmental coverage of the Atlantic Forest provided by biodiversity data is still missing for most groups. Recent initiatives to streamline data on species occurrences, populations, and communities of the Atlantic Forest (Galetti & Ribeiro, 2018) offer a new opportunity to scrutinize the extent of these shortcomings in biodiversity sampling for this biome. Such assessment is key for pinpointing the limitations of current biodiversity data, accounting for them when evaluating the impact of forest fragmentation on biodiversity, and planning future surveys. Determining extinction trends resulting from this kind of information is important to support informed decision-making that becomes particularly important in the context of assessing the potential impacts of eventual changes to the Brazilian Forest Code (Metzger, 2010; Brancalion et al., 2016).

The spatial over aggregation and the dearth of well-sampled sites in fragments located far from cities and major roads is an accessibility bias that is common for most biodiversity taxa (Dennis & Thomas, 2000; Kadmon, Farber & Danin, 2004; Hortal, Lobo & Jiménez-Valverde, 2007; Boakes et al., 2010; Tessarolo et al., 2014; Monsarrat, Boshoff & Kerley, 2018; Stropp et al., 2020). Although the proximity to cities and roads poses a potential threat to biodiversity (Benítez-Lopez, Alkemade & Verweij, 2010), the trend of research teams to choose more accessible places for sampling is indubitable. Most of the available databases are composed of scientific publications originated from theses and research from academic activities. Once these scientific projects have short-term and low funding, the choice for study areas near institutions often guarantees their accomplishment under these conditions. Yet, in the specific case of the fruit-feeding butterfly database, the nearness of the researchers group for Lepidoptera studies also explains the geographical bias on Atlantic Forest (Santos et al., 2018). In addition, butterfly surveys have been historically carried out seeking to sample as many species as possible. This may have caused that larger and more connected fragments were more sampled, following the logic of the species–area relationship (see below). In the Atlantic Forest, large fragments are spatially clustered, which explains the aggregation of fruit-feeding butterfly surveys. An effective plan for filling the sampling gaps from a geographical perspective should consider consulting the geographic distribution of the records in the databases before choosing locations for future expeditions. Unfortunately, the success of this strategy does not depend solely on technical practices. Logistic factors such as funding for research and people engagement are also at stake. Given that science and politics are not on the same page in the current Brazilian scenario, expectations of developing such approach soon in the Atlantic Forest are currently low.

Reaching a sound understanding of the carrying capacity of progressively more fragmented landscapes requires good-quality data on population and community trends from fragments located across the entire environmental, spatial, and habitat gradients. Our results showed a lack of well-sampled sites for fruit-feeding butterflies in small and disconnected forest fragments. The naturalists or researchers behind the historical butterfly expeditions often had a premise for sampling sites to record as much biological diversity as possible, and uncover new species for science. Thus, their predilection for these sites and the number of inventories in extensive natural forests are not a simple coincidence (see Sastre & Lobo, 2009). In present times, most of these Atlantic Forest landscapes are represented by forest remnants within conservation units. From the perspective of the environmental landscape bias, they represent the most common sampling sites.

In contrast, the small forest fragments or those more subject to the deforestation processes are comparatively less sampled. Nonetheless, nowadays most of the Atlantic Forest landscape is represented by forest fragments of these characteristics (Ribeiro et al., 2009). These small fragments may host species that are deemed to extinction due to the time lag between reduction in the size of forest area and the eventual disappearance of the remnant populations (e.g., Triantis et al., 2010). Therefore, estimating extinction debt and determining how to ameliorate it are crucial challenges of fragmentation effects on biodiversity (Kuussaari et al., 2009). It follows that these landscape-sampling gaps compromise our ability to understand how species respond to changes in habitat size. The lack of well-sampled inventories from small and disconnected forest fragments highlights the importance of assessing the quality and coverage of the available biodiversity data before conducting large-scale analyses in macroecology and conservation (Hortal, Lobo & Jiménez-Valverde, 2007; Rocchini et al., 2011; see also Stropp et al., 2016; Ronquillo et al., 2020; Freitas et al., 2021).

Nonetheless, although the well-sampled sites had a higher overall coverage of climatic gradients than that of landscape characteristics, they also failed in providing a fair representation of rare climatic conditions in all bioregions of the Brazilian Atlantic Forest (see Fig. 2). Climatically rare regions lack good-quality inventories since they are not represented on naturalist expeditions and opportunistic surveys (but see Faith & Walker, 1996; Sastre & Lobo, 2009). Consequently, species restricted to small and rare climate habitats (Ohlemüller et al., 2008; Morueta-Holme et al., 2013) are underrepresented in our analysis, an observation that applies to other biodiversity surveys in the Neotropics in more general (Kamino et al., 2012). This absence of information about species occurring in rare climates may compromise our ability to predict shifts in species distribution as a response to climate change and forest fragmentation (see Kamino et al., 2012; Hortal, Lobo & Jiménez-Valverde, 2012; Guisan et al., 2014).

## Conclusions

Current knowledge on fruit-feeding butterflies may provide an incomplete picture of species responses to landscape transformations in the highly dynamic Atlantic Forest biome. This does not necessarily mean that the current database on fruit-feeding butterflies in the Brazilian Atlantic Forest does not have its merit or is not useful. The fair coverage of climatic gradients and accessible and conserved forest fragments provides a solid basis for designing samplings that prioritize small, disconnected fragments in remote locations. The relationship between deforestation and community decay only will be adequately established at a broad scale when biodiversity data provides an even coverage of both landscape and climate variations in the Atlantic Forest. To accomplish this, feeding biodiversity databases with surveys from regions of less common environmental conditions is necessary. In the specific case of the Atlantic Forest butterfly database, these areas are recognized as disturbed forest fragments.

##  Supplemental Information

10.7717/peerj.11673/supp-1 Supplemental Information 1 Supplemental Figures and Tables

## Additional Information and Declarations

### Data Availability

The following information was supplied regarding data availability: The data are available at Santos et al. (2018). Atlantic butterflies: a data set of fruit-feeding butterfly communities from the Atlantic forests. Ecology: https://esajournals.onlinelibrary.wiley.com/doi/full/10.1002/ecy.2507
