# Supplementary material for: Knowledge gaps hamper understanding the relationship between fragmentation and biodiversity loss: the case of Atlantic Forest fruit-feeding butterflies
Source: PeerJ. 2021 Jun 25;9:e11673. doi: 10.7717/peerj.11673 (PMC8237826; doi:10.7717/peerj.11673)
Supplement: Supplemental Information 1 [file peerj-09-11673-s001.docx]

**Supporting Materials**

**Table S1.** data are obtained from published and/or public sources, namely Santos et al. (2018) and GBIF and SpeciesLink biodiversity information networks. Checklists for well-sampled communities identified in this study through the analysis of inventory completeness.

| **Longitude** | **Latitude** |
| --- | --- |
| -50.36666667 | -29.4 |
| -44.6129358 | -22.432586 |
| -46.94628608 | -23.23589328 |
| -48.8923108 | -25.9818594 |
| -49.1020372 | -26.8707245 |
| -52.4 | -27.05 |
| -50.1744444 | -29.4808333 |
| -50.26666667 | -29.58333333 |
| -50.386914 | -29.423669 |
| -53.838197 | -29.747514 |
| -49.36666667 | -25.3 |
| -51.170618 | -23.306445 |
| -51.42111111 | -25.40222222 |
| -51.94305556 | -23.43555556 |
| -52.85666667 | -22.61833333 |
| -54.66666667 | -27.51666667 |
| -50.17777778 | -25.76027778 |
| -53.84194444 | -25.15444444 |
| -48.82222222 | -27.68583333 |
| -49.117766 | -26.974148 |
| -48.820565 | -26.351116 |
| -49.121083 | -26.496003 |
| -49.842142 | -27.456714 |
| -50.660883 | -27.269972 |
| -44.662222 | -22.375278 |
| -42.94916667 | -22.49305556 |
| -42.77166667 | -22.45388889 |
| -42.60694444 | -22.40583333 |
| -43.20472222 | -22.54416667 |
| -43.00055556 | -22.455 |
| -42.51361111 | -22.28 |
| -43.09055556 | -22.64722222 |
| -43.22916667 | -22.94611111 |
| -43.30111111 | -22.54694444 |
| -42.01416667 | -22.54361111 |
| -42.26666667 | -22.52416667 |
| -42.06694444 | -22.43972222 |
| -39.94888889 | -18.43666667 |
| -40.565 | -19.92916667 |
| -40.53611111 | -20.04416667 |
| -40.09583333 | -18.18361111 |
| -40.08111111 | -19.43638889 |
| -40.56111111 | -20.08222222 |
| -40.87416667 | -19.62333333 |
| -41.06388889 | -19.4675 |
| -40.04555556 | -19.14861111 |
| -41.84416667 | -20.41916667 |
| -42.63333333 | -19.8 |
| -43.998219 | -20.004822 |
| -46.032097 | -22.877483 |
| -46.33333333 | -22.9 |
| -46.4425 | -23.99861111 |
| -46.30388889 | -23.81527778 |
| -52.16666667 | -22.45 |
| -46.93555556 | -23.23111111 |
| -46.99694444 | -23.71416667 |
| -45.33333333 | -23.33333333 |
| -47.10805556 | -22.82194444 |
| -46.89138889 | -22.93194444 |
| -47.05 | -22.93333333 |
| -46.567749 | -23.137418 |
| -47.42861111 | -21.85138889 |
| -48.41861111 | -24.27833333 |
| -46.92361111 | -22.82888889 |
| -47.31888889 | -22.38111111 |
| -49.50972222 | -20.825 |
| -49.58916667 | -20.80638889 |
| -45.53444444 | -22.72611111 |
| -48.51666667 | -22.83333333 |
| -46.8 | -21.46666667 |
| -47.531903 | -22.421086 |
| -47.28166667 | -21.32222222 |
| -47.271435 | -20.664522 |
| -46.8 | -22.43333333 |
| -47.93333333 | -25.05 |
| -44.844806 | -23.347353 |
| -45.100175 | -23.328222 |
| -45.17611111 | -23.47861111 |
| -47.91666667 | -21.23333333 |
| -45.52305556 | -22.83833333 |
| -45.49277778 | -23.03694444 |
| -39.71944444 | -13.59527778 |
| -39.89222222 | -18.09027778 |
| -39.57916667 | -15.71222222 |
| -39.52333333 | -15.38111111 |
| -40.4225 | -17.12694444 |
| -39.088672 | -15.173781 |
| -39.58583333 | -16.99861111 |
| -38 | -12.25 |
| -35.70916667 | -9.606111111 |
| -35.86888889 | -8.995 |
| -34.94722222 | -8.010833333 |
| -35.03666667 | -7.964444444 |
| -52.417097 | -31.676944 |
| -43.8333 | -14.9333 |
| -49.726016 | -24.305574 |
| -50.15833333 | -25.09944444 |
| -43.96888889 | -20.01027778 |
| -47.89666667 | -22.26055556 |
| -47.10638889 | -22.29916667 |
| -47.80695556 | -21.60105556 |
| -47.625408 | -21.623477 |
| -47.461075 | -20.240917 |
| -48.16666667 | -23.58333333 |
| -50.13333333 | -22.66666667 |
| -41.25027778 | -15.51388889 |
| -50.611356 | -29.44282 |
| -52.432475 | -31.814044 |
| -49.853506 | -29.364375 |
| -51.816454 | -20.993649 |
| -53.889069 | -27.197939 |
| -51.026006 | -30.369561 |
| -51.111174 | -29.559801 |
| -51.157301 | -30.098678 |
| -46.57322491 | -21.76992582 |
| -45.94766311 | -21.42964807 |
| -39.11666667 | -12.65 |


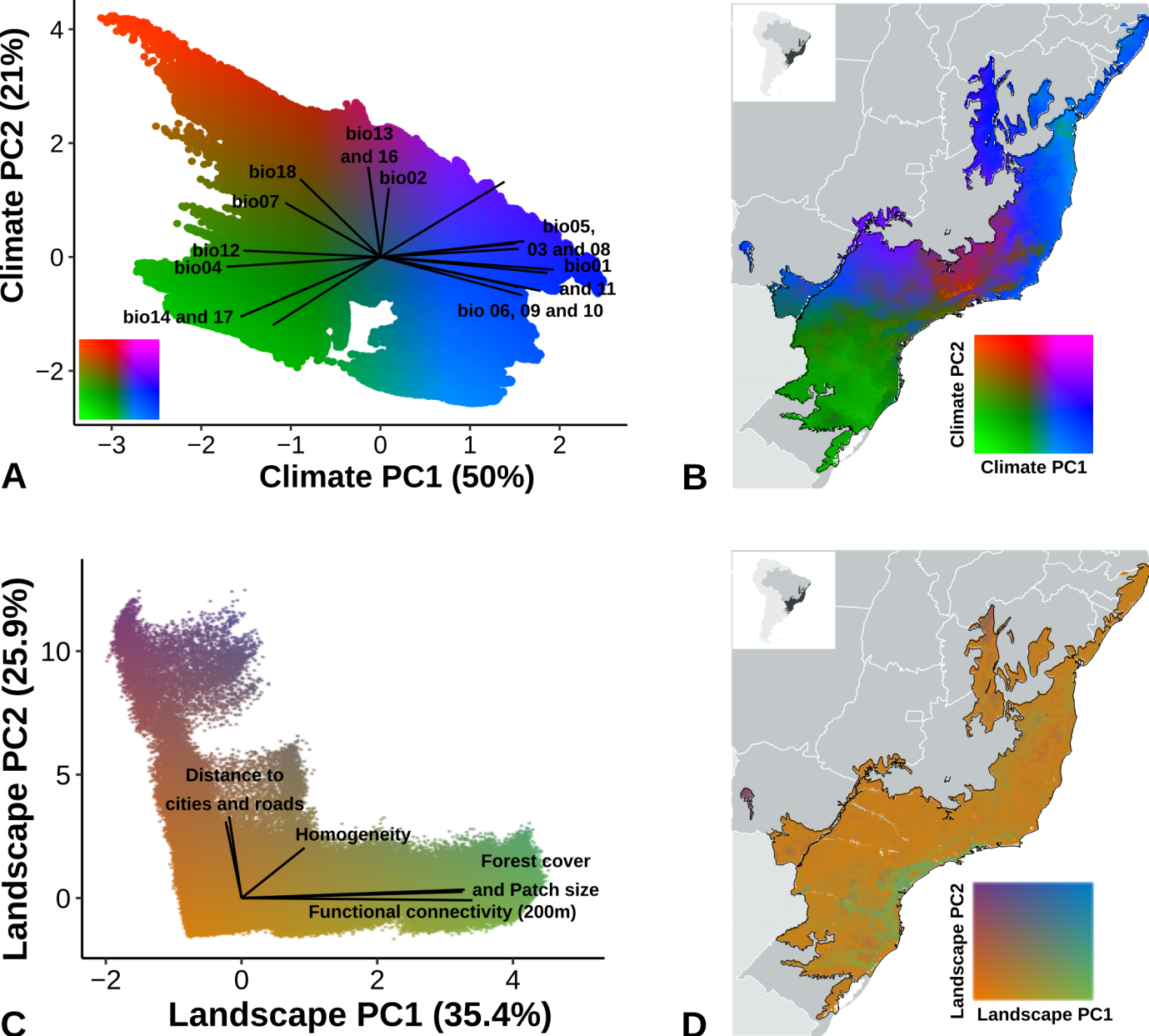


**Fig S1.** Results of PCA analyses, including the location of all Brazilian Atlantic Forest cells in the PCA axes, and the relationships of the vectors of the original variables with these axes. (a) climate variables (Bio01 – Bio19); (b) landscape metrics (200m functional connectivity, forest cover, patch size, homogeneity, distance to roads, distance to cities).

**Table S2.** The first four axes of both Principal Component Analysis (PCA) of climate (19 variables extracted from Fick & Hijmans, 2017) and landscape (five variables extracted from the Spatial Ecology and Conservation Laboratory, Ribeiro pers. comm.), and their respective scores. (SD = Standard deviation).

| **Variables** | **PC1** | **PC2** |
| --- | --- | --- |
| **Climate** |  |  |
| Annual Mean Temperature | 0.932 | -0.141 |
| Mean Diurnal Range (Mean of monthly (max temp − min temp)) | 0.047 | 0.606 |
| Isothermality (Mean Diurnal Range/Temperature Annual Range) (×100) | 0.803 | 0.139 |
| Temperature seasonality (SD × 100) | -0.858 | -0.086 |
| Max Temperature of Warmest Month (MTWM) | 0.757 | 0.119 |
| Min Temperature of Coldest Month (MTCM) | 0.895 | -0.299 |
| Temperature Annual Range (MTWM‐MTCM) | -0.531 | 0.475 |
| Mean Temperature of Wettest Quarter | 0.772 | 0.072 |
| Mean Temperature of Driest Quarter | 0.794 | -0.337 |
| Mean Temperature of Warmest Quarter | 0.770 | -0.264 |
| Mean Temperature of Coldest Quarter | 0.967 | -0.112 |
| Annual Precipitation | -0.765 | 0.055 |
| Precipitation of Wettest Month | -0.069 | 0.792 |
| Precipitation of Driest Month | -0.779 | -0.526 |
| Precipitation Seasonality (Coefficient of Variation) | 0.693 | 0.661 |
| Precipitation of Wettest Quarter | -0.067 | 0.785 |
| Precipitation of Driest Quarter | -0.781 | -0.524 |
| Precipitation of Warmest Quarter | -0.448 | 0.685 |
| Precipitation of Coldest Quarter | -0.602 | -0.600 |
| *SD* | 9.516 | 4.008 |
| *Proportion of Variance* | 0.501 | 0.211 |
| *Cumulative Proportion* | 0.501 | 0.712 |
| **Landscape** |  |  |
| Proportion of habitat | 0.814 | 0.064 |
| Fragment size | -0.058 | 0.774 |
| Functional Connectivity | 0.851 | -0.023 |
| Homogeneity | 0.232 | 0.508 |
| Distance to urban areas | 0.822 | 0.086 |
| Distance to roads | -0.046 | 0.828 |
| *SD* | 2.121 | 1.555 |
| *Proportion of Variance* | 0.354 | 0.259 |
| *Cumulative Proportion* | 0.354 | 0.613 |
